# Case Report: Diagnosis and surgical procedure of an ovarian teratoma in a green iguana (*Iguana iguana*)

**DOI:** 10.3389/fvets.2026.1970094

**Published:** 2026-10-23

**Authors:** Virginia Zdzuj, Johannes Hetterich, Christina Puff, Michael Pees

**Affiliations:** 1 Department of Small Mammal, Reptile and Avian Medicine and Surgery, University of Veterinary Medicine Hannover, Hanover, Germany; 2 Department of Pathology, University of Veterinary Medicine Hannover, Hanover, Germany

**Keywords:** case report, coeliotomy, iguana, ovarian disease, teratoma

## Abstract

A fourteen-year-old, 3.9 kg, intact female green iguana (Iguana iguana) was presented to a veterinary clinic with an 8-week history of persistent apathy and progressive bilateral coelomic distension. Physical examination revealed poor body condition and significant deterioration of the general condition. The caudal coelom was circumferentially distended and palpation revealed multiple firm, irregularly shaped intracoelomic structures. Radiographic evaluation showed a large radiopaque mass in the median and caudal coelom. Ultrasonography confirmed a large intracoelomic mass, high-grade ascites, and multiple smaller hyperechogenic masses within the caudal coelom. Microscopic examination of the ascites fluid revealed cocci, heterophilic granulocytes, and degranulated mast cells. Based on the findings of all examinations, differential diagnoses included severe follicular stasis, multiple intracoelomic abscesses, neoplastic disease, and concurrent coelomitis. Subsequently, a lateral exploratory coeliotomy was performed under general anesthesia. A 5 x 4.5 x 4.5 cm intracoelomic mass and multiple irregular, ovoid, rough, whitish masses measuring approximately 2 x 3 x 2.5 cm were surgically removed. The animal failed to recover from anesthesia and died 3 h postoperatively. Histopathological examination revealed a teratoma and a severe, multifocal, lipogranulomatous oophoritis. This case report describes and characterizes a neoplastic disease that has rarely been reported in iguanas. Nevertheless, ovarian neoplasia should be considered an important differential diagnosis for common reproductive disorders in female lizards.

## 1 Introduction

Neoplastic diseases in reptiles have been described with an increasing frequency, although they were once considered less common (1, 2). The apparent rise in the prevalence of neoplasia in reptiles may be associated with an increased longevity of pet reptiles, enhanced veterinary diagnostic services and expertise and greater awareness among reptile owners, resulting in more frequent presentation of affected reptiles (1, 3). Also, genetic predisposition, environmental factors, and viral etiologies affect the development of neoplastic disease in reptiles (1). Reported prevalence data on neoplasia in reptiles vary considerably, reflecting differences in study populations and data sources (1, 2). A large-scale retrospective study investigating neoplastic diseases found a prevalence of 2.63 % among all lizards presented in a specialized teaching hospital (1). In contrast, another retrospective analysis of histopathological and cytological diagnostic samples from a large population of clinically affected lizards found a prevalence of 22.9% (2). This study also documented numerous neoplasms in iguanas, including ovarian adenocarcinoma (2). Teratomas have been described sporadically in lizards (4). These tumors are composed of tissues derived from two or more germ cell layers, including the ectoderm, mesoderm, and endoderm (5). Tumors can consist of a variety of tissues including cartilage, nervous tissue, adipose tissue, hair follicles, respiratory epithelium, sebaceous glands, and apocrine glands (6). Teratomas most commonly arise in the gonads, such as the ovaries and testes, but can also occur at extragonadal sites such as the central nervous system, sacrum, or mediastinum (7). Numerous reports have described ovarian neoplasms in iguanas, including a benign teratoma (8), malignant teratomas (9, 10), an adenocarcinoma (11), and a papillary cystadenocarcinoma (12). Radiography, ultrasonography and histopathology represent the diagnostic modalities most commonly used for tumor verification (2, 8–10, 13). When treatment is pursued, complete surgical excision is considered the primary therapeutic approach for teratomas (8–10).

The primary ovarian lesion in the present case was a teratoma. However, unlike previously reported cases of ovarian teratomas in iguanas, it was accompanied by severe, multifocal lipogranulomatous oophoritis as an additional and unusual pathological lesion.

## 2 Clinical case

### 2.1 History and clinical examination

A fourteen-year-old, 3.9 kg, intact female iguana (Iguana iguana) raised under human care was presented with an 8-week history of anorexia, apathy and progressive bilateral coelomic distension (Figure 1). The iguana had been housed individually in an 18 m2 glass enclosure for 14 years. The temperature ranged between 22 °C (72 °F) to 45°C (113 °F). The animal was fed daily with basil, bok choy, onion, leek, parsley and dried food (ad libitum). The iguana was presented annually in spring for routine health examinations. Physiological follicle formation was repeatedly observed. However, oviposition had never been reported by the animal owner.

**Figure 1:**
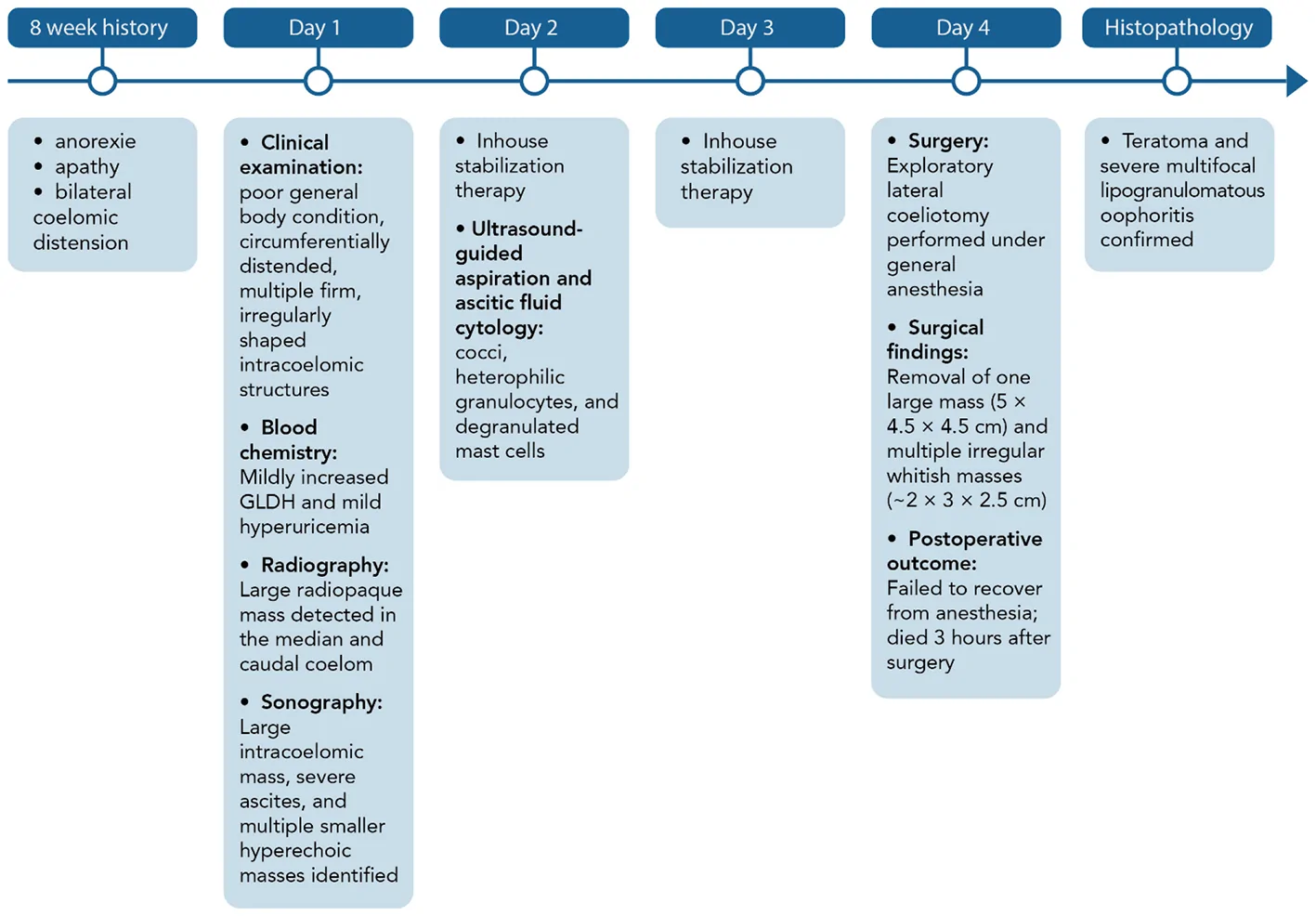
Timeline of the clinical signs, diagnostic investigations, and interventions performed throughout the case.

Clinical examination revealed marked deterioration of the general condition and a poor body condition. The caudal coelom was circumferentially distended, and palpation identified multiple firm, irregularly shaped intracoelomic structures. Differential diagnoses included severe chronic follicular stasis, multiple intracoelomic abscesses, and neoplasia involving various coelomic organs.

### 2.2 Diagnostic procedure

Initial diagnostic methods included blood chemistry examination, radiography and ultrasonography (Figure 1). Blood samples were collected from the ventral tail vein under manual restraint. Glutamate dehydrogenase (GLDH) activity appeared to be mildly increased (12.1 U/L). However, this finding should be interpreted with caution, as validated species-specific reference intervals for GLDH in green iguanas are currently lacking. Mild hyperuricemia (URIC: 7.81 mg/dl; reference range: 0.9–6.7 mg/dl) was also detected (14). Dorsoventral and horizontal beam lateral radiographic projections were obtained under manual restraint (Figure 2; digital X-ray; detector system: Fujifilm Console Advance DR-ID 300 CL, Fujifilm Europe GmbH, Düsseldorf, Germany; tube system: Gierth X-ray International GmbH, Riesa, Germany; film focus distance FFD 60 cm, 55 kV, 5 mAs). In both projections, the median and caudal coelom exhibited marked soft tissue dense opacity with a concurrent loss of regular coelomic detail.

**Figure 2:**
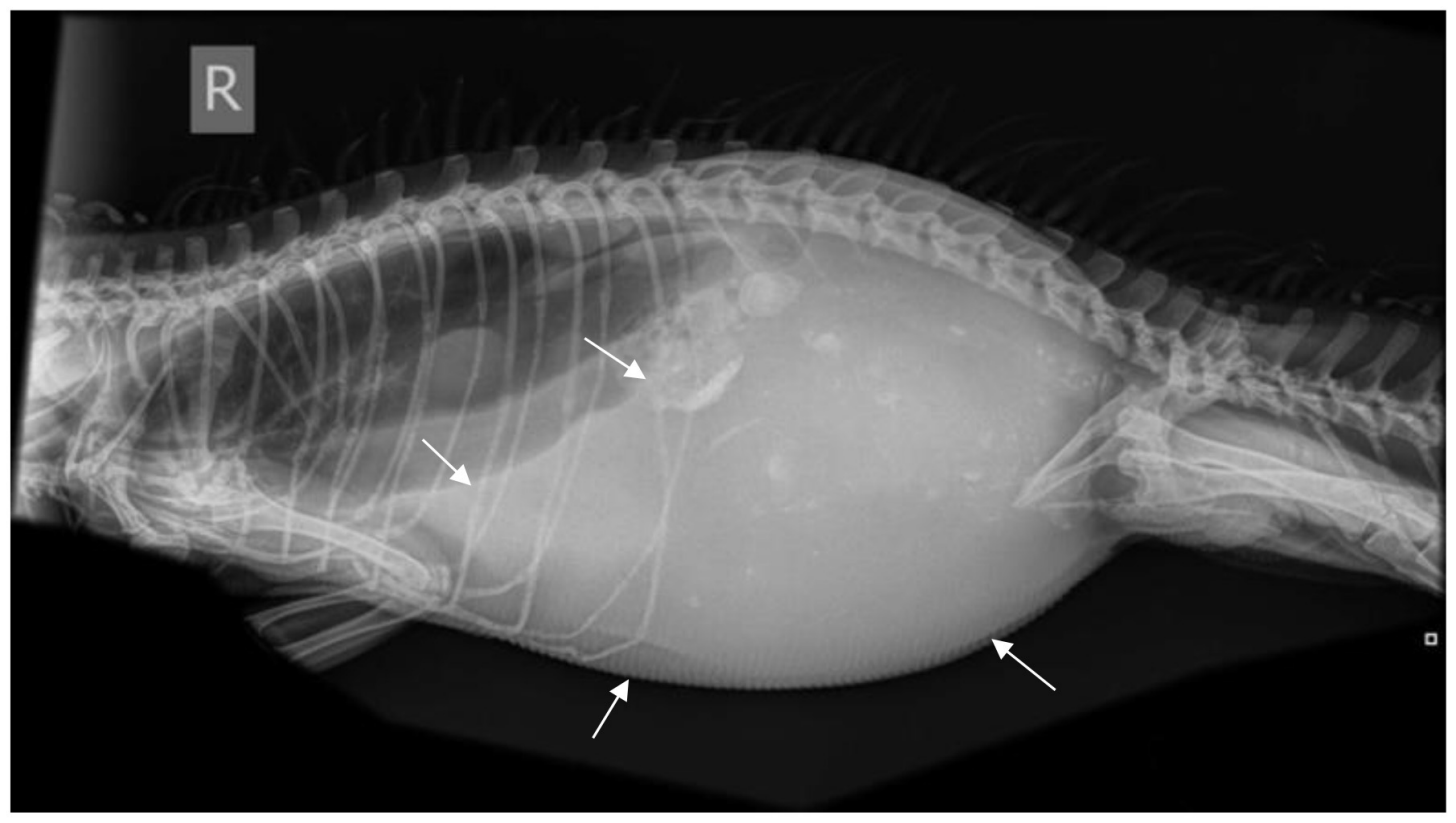
Laterolateral radiograph of a fourteen-year-old green iguana (Iguana iguana), obtained in a left-to-right projection. A marked soft tissue dense opacity and a coelomic distension in the median and caudal coelom is clearly visible. Arrows indicate the marked soft tissue opacity.

Ultrasonographic examination was performed under manual restraint with the iguana positioned in dorsal recumbency (Micro curved array transducer, 5–9 MHz; Vivid 7 Dimension; GE Healthcare GmbH, Solingen, Germany). Transverse and longitudinal scans obtained from a ventral coupling site revealed a large volume of free intracoelomic fluid containing multifocal, moderate corpuscular elements. Multiple hyperechogenic, heterogeneous and partially vascularized masses were identified in the caudal coelom (Figure 3).

**Figure 3:**
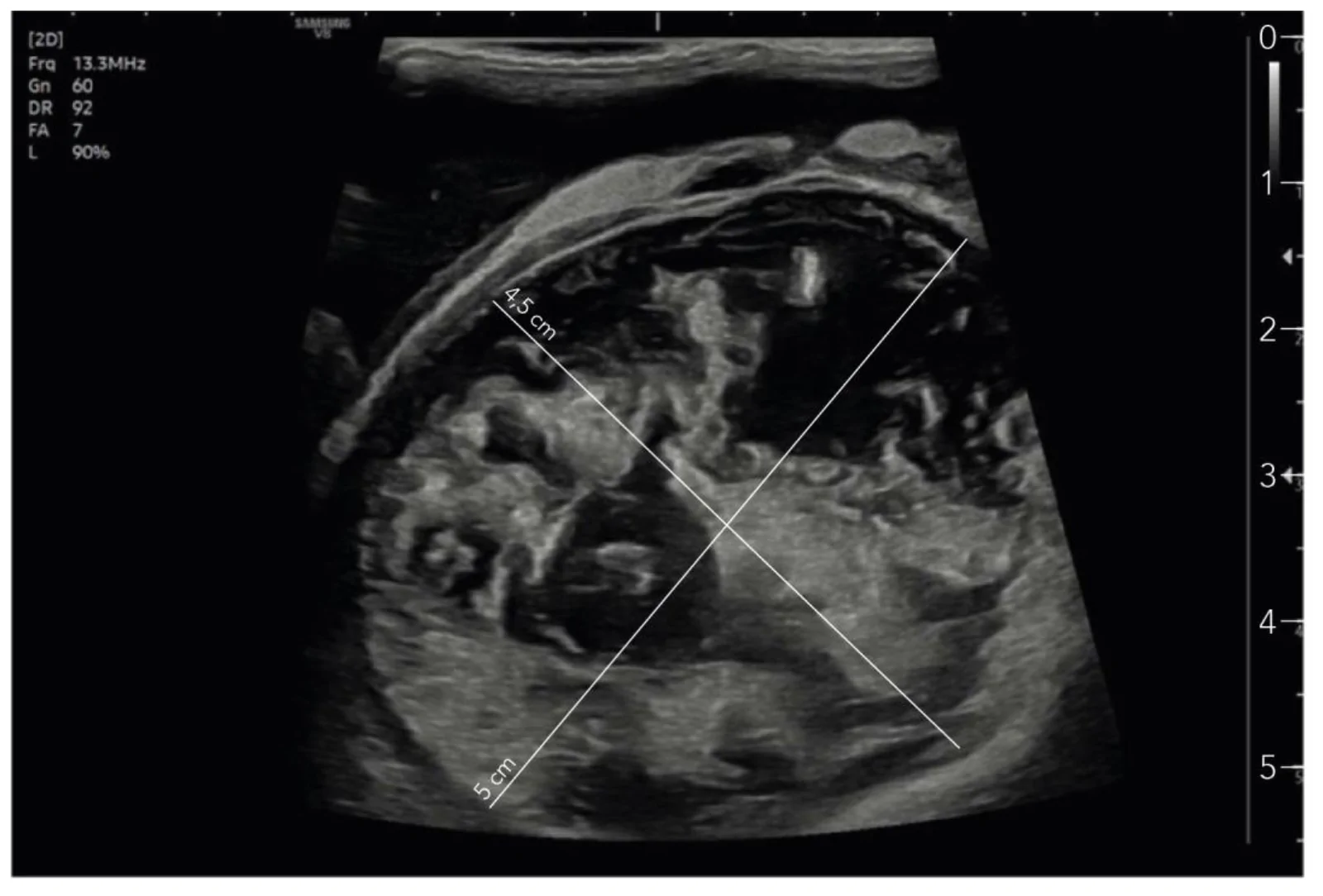
Longitudinal ultrasonographic image of the caudal coelom in a green iguana (Iguana iguana) acquired with a 5 to 9 MHz micro curved array transducer (penetration depth 6 cm; frequency 13.3 MHz) showing an 5 x 4,5 cm, round, irregular intracoelomic mass. Due to the focus on the detailed representation of the tumor structures, the tumor borders are not fully visible in their entirety in the image.

Based on the ultrasonography findings, neoplasia was considered the primary differential diagnosis. At that time, the organ of origin could not be clearly identified. As an additional diagnostic procedure, an ultrasound-guided coelomic centesis was performed through a caudolateral coupling site of the left coelom. A total of 200 mL of brownish, serous fluid was aspirated. The procedure was repeated once daily for three consecutive days, yielding 200 mL of fluid each time. An aliquot of the fluid was stained using DiffQuick (LT-SYS, color solution I +II, Labor + Technik Eberhard Lehmann, Berlin, Germany; methanol / methylene blue, Labor + Technik Eberhard Lehmann, Berlin, Germany) and submitted for cytological evaluation. Microscopic examination revealed cocci, heterophilic granulocytes, and occasional degranulated mast cells. Fluid analysis showed a total protein concentration of 3.55 g/dl, albumin of 1.1 g/dl and urea of 7 mg/dl. These findings were considered consistent with an inflammatory process within the coelomic cavity. The animal was hospitalized for preoperative stabilization and received supportive therapy including fluid administration (15 mL/kg IV SID, Sterofundin, ISO 1/1 E, B. Braun Melsungen AG, Melsungen, Germany), analgesia (0.3 mg/kg SC SID, Meloxicam; Metacam, Boehringer Ingelheim GmbH, Ingelheim, Germany), and antibiotic treatment (10 mg/kg SC SID, Marbofloxacin; Marbocyl FD 1%, Vetoquinol, Ismaning, Germany). Hepatic and renal support was provided using phytotherapeutic medications (0.5 mL SC SID, Hepar comp. PlantaVet, Bad Waldsee, Germany; 50 mg/kg PO SID, LegaPhyton 200, Vetoquinol, Ismaning, Germany; 0.5ml SC SID, Renes/Viscum comp. PlantaVet, Bad Waldsee, Germany). The animal was maintained within its preferred optimal temperature zone (POTZ) for 3 days, with no clinical improvement observed during hospitalization. An exploratory coeliotomy was subsequently performed.

### 2.3 Coeliotomy

General anesthesia was induced with hydromorphone (1 mg/kg IM; Hydromorphon 2 mg/mL, hameln pharma GmbH, Hameln, Germany), alfaxalone (5 mg/kg IV; Alfaxan Multidose 10 mg/mL, Jurox Pty Limited, 85 Gardiner Street, Rutherford NSW 2320, Australia), ketamine (10 mg/kg IV; Ketamin 100 mg/mL, CP-Pharma Handelsgesellschaft mbH, Burgdorf, Germany) and medetomidine (0.1 mg/kg IV; Cepetor 1 mg/mL, CP-Pharma Handelsgesellschaft mbH, Germany). The iguana was intubated with a modified 9.5 feeding tube. Anesthesia was maintained with isoflurane (1–2%) and oxygen (0.8 L/min) delivered via intermittent positive pressure ventilation (IPPV). Anesthesia monitoring included heart rate assessment using a Doppler device and continuous measurement of cloacal body temperature. During coeliotomy, the lizard was kept warm using a heating mat and supplemental heating pads. The iguana was positioned in lateral recumbency and aseptically prepared using an iodine solution (Jodosept^®^, Vetoquinol GmbH, Ismaning, Germany) and chlorhexidine. A transverse skin incision was made on the left flank using a No. 11 scalpel blade. The underlying musculature and peritoneal membrane were incised stepwise using Metzenbaum scissors. Upon entering the coelomic cavity, approximately 400 mL of brownish, serous ascites fluid was aspirated. Due to limited accessibility of the coelomic mass, a cranial relieving incision was additionally performed, allowing improved exposure and subsequent complete resection of the tumor. Extensive fibrinous deposits were also removed from the coelomic membrane. The ovarian mass (5 x 4.5 x 4.5 cm) was characterized by solid, firm, and nodular areas. The vascular supply to the tumor was ligated using bipolar electrocautery, and the mass was completely excized (Figures 4, 5). Multiple irregular, ovoid, firm, whitish masses (approximately 3 x 2 x 2,5 cm) were multifocally distributed within the coelomic cavity. Several masses were adherent to the mesentery and coelomic membranes. Except for one mass lesion inseparably attached to the greater curvature of the stomach, all masses were dissected using bipolar electrocautery and scissors. After removal of all visible masses, the coelomic cavity was lavaged with 200 mL of warm isotonic crystalloid solution (Sterofundin ISO 1/1 E) and subsequently suctioned. The coelomic organs were thoroughly inspected with no additional masses identified. Closure was performed in a stepwise standard closure of the coelom using a continuous appositional suture pattern for the peritoneal membrane and abdominal musculature (absorbable coated polyglycolic acid), followed by simple interrupted skin sutures using the same material.

**Figure 4:**
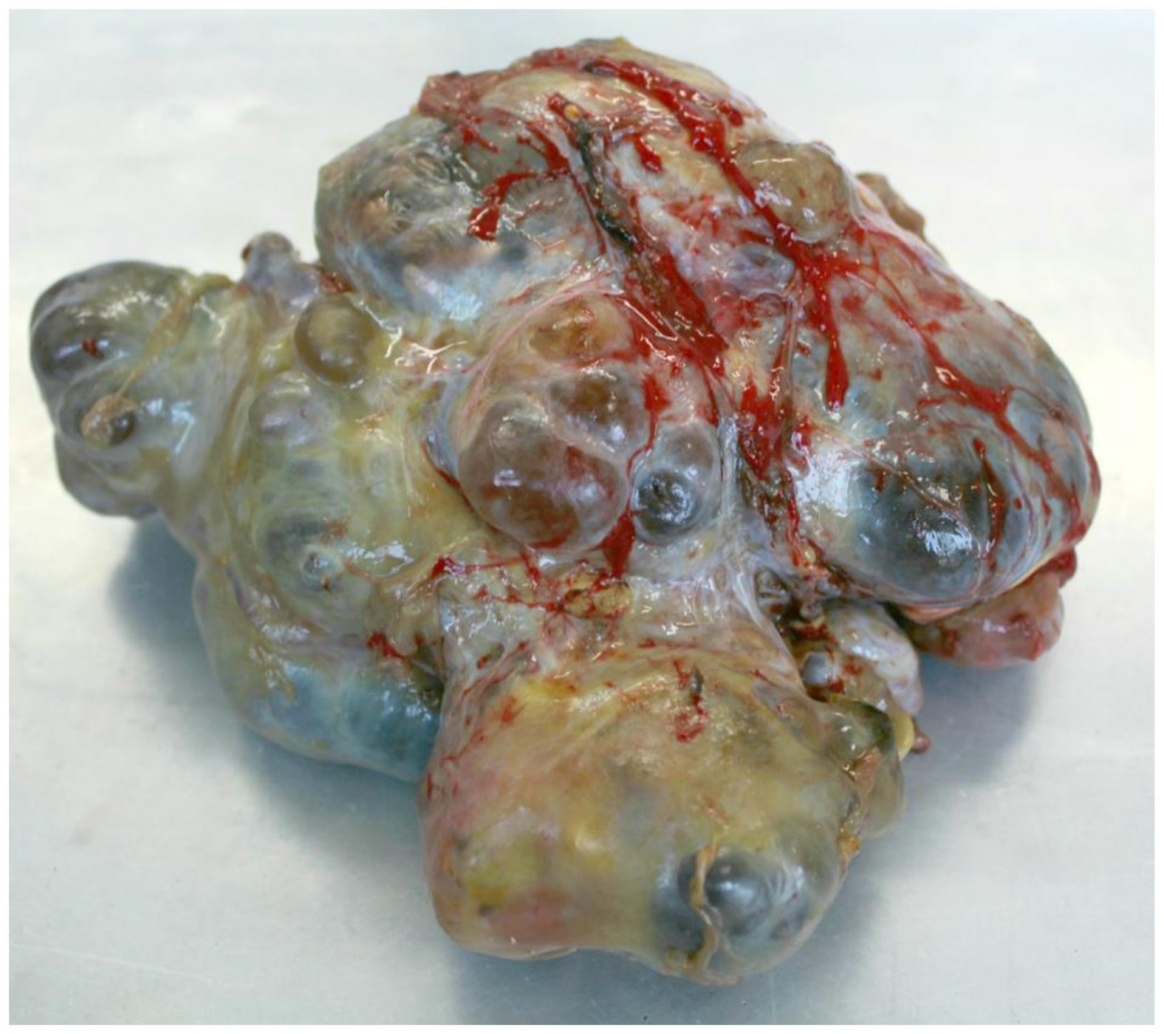
A multilobular ovarian mass measuring 5 x 4.5 x 4.5 cm, characterized by solid, firm, and nodular areas, was excised.

**Figure 5:**
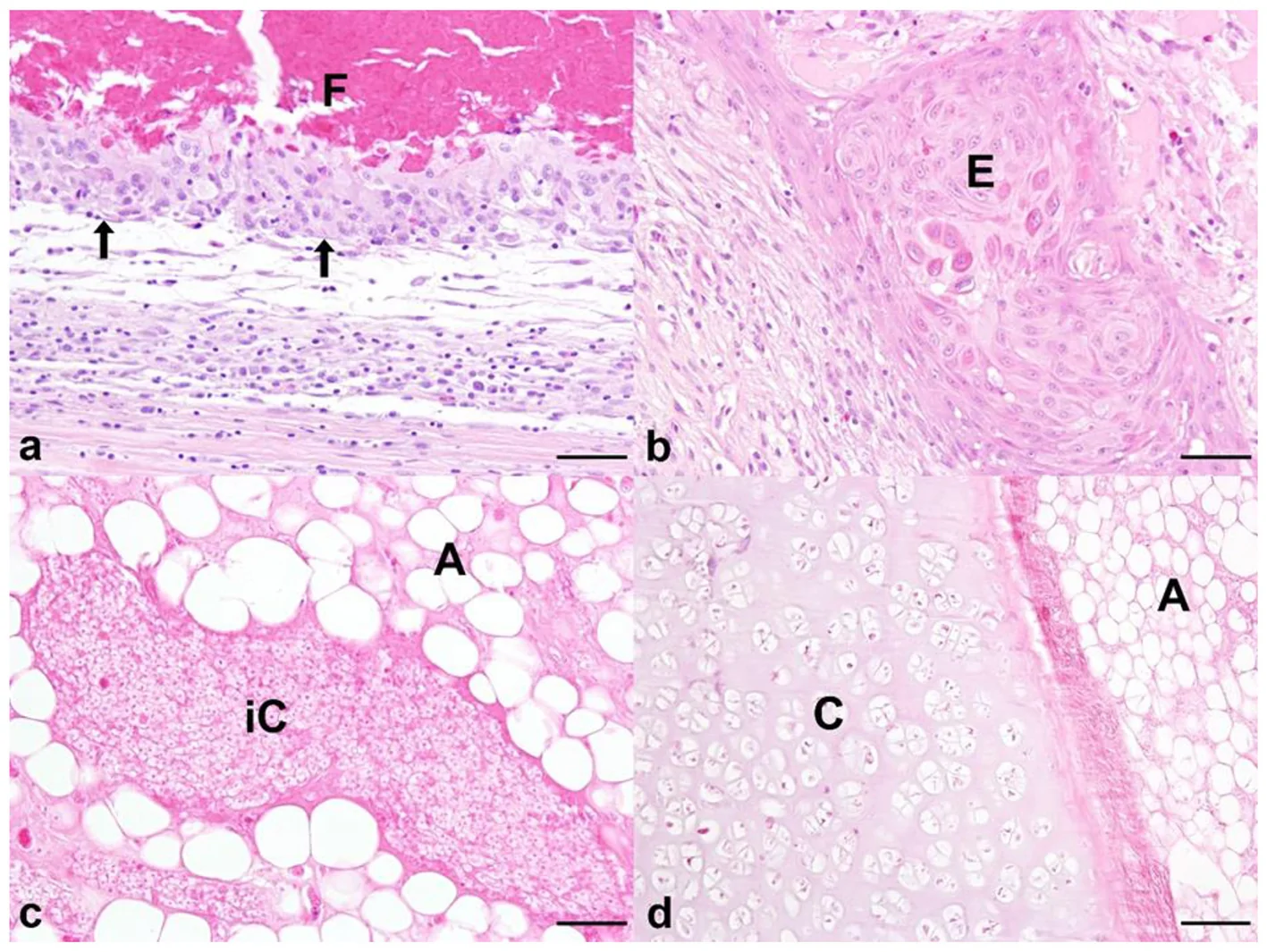
**(a)** The ovary displayed a severe lipogranulomatous inflammation with macrophages and epitheloid cells (arrows) surrounding follicular proteinaceous material (F). The teratoma was composed of several tissue types. **(b)** These tissues included partially keratinizing epithelial cells (E). **(c)** Furthermore, the tumor contained immature cartilage (iC) and adipose tissue (A). **(d)** In other areas the neoplasm was composed of mature cartilage **(C)** and adipose tissue (A). Hematoxylin and eosin. Bars 50 μm (a–c) and 100 μm **(d)**, respectively.

Postoperatively, the iguana did not regain spontaneous respiration. Intermittent positive pressure ventilation was maintained for a total of 3 h. Continuous heart rate monitoring was performed, and the lizard was kept within an appropriate temperature range. Despite supportive care, cardiac activity ceased, and the animal died approximately 3 h postoperatively.

### 2.4 Histopathological examination

The excised mass was fixed in 10% neutrally buffered formalin, routinely processed and sections were stained with hematoxylin and eosin. The sample consisted of remaining ovarian tissue exhibiting a multifocal severe infiltration with macrophages, epitheloid cells and fewer lymphocytes often arranged around eosinophilic proteinaceous material (most likely follicular protein; Figure 5a) and a multinodular mass. This mass consisted of different tissue types organized in a haphazardly arranged pattern. The tissues identified included nests and islands of partially keratinizing epithelium (Figure 5b), which multifocally formed central keratin-filled cysts, immature (Figure 5c) and mature cartilage (Figure 5d), adipose tissue (Figure 5d) and fibrous connective tissue. The tissues exhibited a mild anisocytosis and anisokaryosis and a low mitotic count with less than one mitotic figure per 2,37 mm^2^. Taken together an ovarian teratoma and a severe, chronic, multifocal lipogranulomatous oophoritis were diagnosed (Figure 1).

## 3 Discussion

Historically, neoplastic disease in reptiles was considered less common compared with mammals (15). However, more recent studies have reported higher incidences of neoplasia in reptiles (1, 2). Over the past two decades, the number of published clinical and post-mortem case reports, as well as retrospective evaluations, has increased considerably (16). This trend may reflect improved diagnostic capabilities (16), and a more adequate management and husbandry of captive reptiles leading to an increased longevity. Since neoplasia is more frequently observed in older reptiles (3), increased lifespan in captivity may contribute to higher reported incidence. Reported tumor prevalence rates range from approximately 2 to 3% (1, 16, 17). Neoplastic disease is described to be more common in snakes, followed by lizards, chelonians and crocodiles (4). Another study focusing on lizards found neoplastic disease in 22.9% of cases (2), based on 690 samples of clinically affected animals. Substantial differences in study populations, sample types and levels of methodological standardization likely account for the wide variation in reported prevalence rates.

As in mammals and birds, teratomas are considered uncommon in reptiles (18–22). In a study on reptilian neoplasia (4), a prevalence of 1.2% was reported for teratomas in lizards. Teratomas most commonly arise in the reproductive tract, particularly the ovaries and testes (23). Several case reports of teratomas in reptiles have been published to date (8, 9, 22, 24–26). In addition to the green iguana (Iguana iguana), in which both benign (8) and malignant (9) teratomas have been described, cases have also been reported in other reptile species, including the Fiji island banded iguana (Brachylophus fasciatus) (8), garter snake (Thamnophis sirtalis) (25), red-eared slider turtle (Trachemys sripta elegans) (22, 26) and bearded dragon (Pogona vitticeps) (24).

The iguana in the present report was presented annually for routine health examinations, which regularly included a physical examination and a fecal examination. Blood biochemistry, radiography, and ultrasonography had been performed infrequently. 9 months elapsed between the last health examination and the presentation due to clinical symptoms. In this case, it remains unclear whether the absence of oviposition was associated with dystocia and whether dystocia occurred prior to tumor emergence or the tumor growth, with its associated space-occupying effect, led to follicular stasis. Interestingly, recurrent dystocia has previously been reported in a green iguana with a teratoma (8).

The clinical signs observed in the present case partially overlap with those reported in previous iguana cases, including anorexia, apathy and bilateral coelomic distension (8, 9, 12, 21). In female reptiles with similar clinical signs, follicular stasis is commonly considered a differential diagnosis (9). In another case described, follicles were detected before the teratoma was diagnosed. The follicles subsequently increased in size, with no subsequent oviposition observed (21). However, these clinical findings are non-specific and may also be associated with other diseases in reptiles, such as renal disease, dystocia or viral infections (15, 27). Consequently, further diagnostic work-up is essential to differentiate between potential etiologies.

Based on the differential diagnoses after the diagnostic evaluation in the present case, a neoplastic process was considered most likely, as follicular stasis typically presents with a different morphological pattern. The combined radiographic and ultrasonographic findings supported the decision to perform an exploratory coeliotomy. Radiography and/or ultrasonography have also been employed in previously published cases with an ovarian teratoma (8, 9, 21). Additional diagnostic imaging, such as a magnetic resonance imaging (MRI), might have provided a more precise characterization of the intracoelomic pathology and improved surgical planning. However, it would not have altered the overall treatment strategy to proceed with a coeliotomy in this patient.

The existing literature indicates that ovarian teratomas are characterized histologically by a heterogeneous composition of variously differentiated tissue types (9, 21). A distinctive feature of the present case was the concurrent occurrence of pronounced, chronic, multifocal lipogranulomatous oophoritis as a concomitant lesion associated with the ovarian teratoma.

According to the literature, differential diagnoses for ovarian neoplasia in reptiles include benign and malignant teratomas (8–10, 21), adenocarcinomas (11), papillary cystadenocarcinomas (12), and granulosa cell tumors (4). The marked ascites observed in the present case may have resulted from an inflammatory process secondary to pressure-induced yolk release or follicular rupture (15). Ascites has also been documented in several cases previously described (9, 12). Free yolk within the coelomic cavity has been described as a bright yellow, viscous fluid, whereas progression to yolk coelomitis may result in an inflammatory, hemorrhagic, or purulent coelomic effusion (28, 29). In the present case, the brownish, serous character of the coelomic fluid, together with the presence of cocci, heterophilic granulocytes, and occasional degranulated mast cells, supported an inflammatory process within the coelomic cavity. The detection of urea in the coelomic fluid may indicate contamination with urinary material. However, the comparably low urea concentration does not allow a definitive conclusion regarding the origin of the fluid.

In the management of teratomas, surgical resection is recommended due to their potential for rapid growth, which may result in displacement or compression of adjacent intracoelomic organs (8). In previously published case reports, surgical resection of the tumor has proven to be effective and life-prolonging (8, 9).

In the present case, the owners had observed progressive circumferential coelomic enlargement for 8 weeks prior to presentation. At the initial examination, the animal already showed a marked deterioration in general condition. Both hospitalization and surgical intervention were therefore considered high-risk, with a guarded prognosis for anesthetic recovery. Given the severely compromised preoperative condition, euthanasia would have been a reasonable alternative.

The iguana's postoperative death was considered most likely to have resulted from multifactorial circulatory failure with a potentially hypovolemic component (fluid and protein loss). Potential contributing factors included perioperative fluid loss, despite fluid therapy, as well as the animal's markedly compromised general condition and physical deterioration, including the concurrent inflammatory process. Prior to surgery, a total of approximately 600 mL of coelomic fluid had already been removed from the 3.9 kg iguana, representing approximately 154 mL/kg body weight. Given the animal's marked deterioration and potential hypovolemia, the additional cardiovascular stress associated with anesthesia may have contributed to circulatory instability and the fatal outcome. However, the exact underlying mechanism of the circulatory failure could not be determined retrospectively.

In summary, the present report describes a 14-year-old female iguana diagnosed with a rare ovarian teratoma accompanied by severe, multifocal, lipogranulomatous oophoritis. This case highlights the importance of including neoplasia in the differential diagnosis of reptiles with non-specific signs, such as coelomic distension. It also emphasizes the need for timely diagnostic evaluation and intervention to optimize patient management and outcome.

## Data Availability

The original contributions presented in the study are included in the article/supplementary material, further inquiries can be directed to the corresponding author.
